# Prevalence of Impaired Physical Mobility in Dialysis Patients: A Single-Centre Cross-Sectional Study

**DOI:** 10.3390/jcm12206634

**Published:** 2023-10-20

**Authors:** Špela Bogataj, Jernej Pajek, Blaž Slonjšak, Vanja Peršič

**Affiliations:** 1 Department of Nephrology, University Medical Centre Ljubljana, 1000 Ljubljana, Slovenia; spela.bogataj@kclj.si (Š.B.); jernej.pajek@mf.uni-lj.si (J.P.); blaz.slonjsak@kclj.si (B.S.); 2 Faculty of Medicine, University of Ljubljana, 1000 Ljubljana, Slovenia

**Keywords:** physical mobility, impairment, chronic kidney disease, hemodialysis, morbidity

## Abstract

Impaired physical mobility in hemodialysis (HD) patients is considered an important modifiable risk factor of increased all-cause morbidity and mortality. To our knowledge, no study to date has determined the overall burden of limited physical mobility in prevalent HD patients. The aim of this research is to investigate impaired physical mobility and its clinical correlates. We conducted a cross-sectional observational study in all patients of the Centre for Acute and Complicated Dialysis at the University Medical Centre of Ljubljana, where the most complex patients receive HD on average three times per week. The data were collected through interviews based on a prepared questionnaire and medical history review. A total of 205 patients were included in this study (63.9 ± 15.4 years). Sixty percent (122/205) of the patients had little or no physical mobility impairment, and others were categorized with a minor or severe mobility limitation. A minor mobility impairment was found in 21% (43/205) of patients: 10 patients (5%) used a mobility aid in the form of a crutch, 9 patients (4%) were dependent on two crutches or a walker, and 24 patients (12%) were temporarily dependent on the assistance of a third person. Severe mobility limitations were observed in 22% (40/205) of patients, ranging from being confined to bed (19/205, 9%), confined to bed but able to perform some movements (19/205, 9%), and ambulatory but dependent on the assistance of a third person for locomotion (2/205, 1%). The most common causes of the limitation of mobility were neurological (19/40, 47.5%), cardiovascular (9/40, 22.5%), musculoskeletal (8/40, 20%), and other causes (4/40, 10%). A significant, moderate positive correlation was observed between mobility problems and the age of the participants (r = 0.36, *p* < 0.001), while a significant, small positive correlation was obtained between the mobility problems and C-reactive protein (r = 0.15, *p* = 0.044). Moreover, mobility problems had a small but significant negative correlation with albumin levels (r = −0.15, *p* = 0.042). When controlling for age, results yield no significant correlations, and, in regression analysis, only the age (*p* < 0.001) and male gender (*p* = 0.007) of the participants were independent predictors of mobility impairment. We conclude that impaired mobility has a high overall prevalence among chronic HD patients. Strategies to prevent and improve mobility limitations are strongly needed.

## 1. Introduction

Chronic kidney disease (CKD) is a condition characterized by the gradual loss of kidney function over time, leading to a decrease in the organ’s ability to filter waste products from the blood and regulate essential bodily functions [1]. Patients with CKD are often less physically active and less physically capable compared to their healthy counterparts [2]. Physical inactivity is thought to be a contributing factor to the increased mortality seen in this population [3]. It can lead to a worsening of other comorbidities such as diabetes, hypertension, and cardiovascular disease [4], and it can have a number of negative consequences for patients with CKD, including muscle wasting, decreased cardiovascular fitness, and impaired mobility [5]. Additionally, a substantial and continuous decline in physical function is noticed at the initiation of dialysis. This decline is a progressive process with further worsening of physical function during hospitalizations and acute illnesses [6]. The final result of the aforementioned deterioration often leads to irreversibly impaired physical mobility with dependence on the caregivers. Recent research [7] has highlighted the importance of understanding the factors influencing mobility decline during the induction phase of dialysis. This phase, which often involves emergency dialysis start, has been associated with a significant decline in walking independence among CKD patients. Therefore, a comprehensive examination of physical mobility in CKD patients is essential for improving their overall quality of life and informing early rehabilitation strategies.

Impaired mobility can be a temporary or permanent condition (it can have both physical and psychological consequences), and it can be caused by a variety of modifiable and non-modifiable risk factors [8]. Besides physical inactivity and the initiation of renal replacement therapy, reduced mobility can have a range of other causes in CKD patients. Musculoskeletal disorders, cardiovascular diseases, neurological disorders, cognitive disorders, and acute illnesses are all common modifiable risk factors that can contribute to impaired mobility [9,10]. Terminal musculoskeletal, cardiovascular, and neurological diseases are common non-modifiable risk factors that can lead to permanent impairment [10,11]. Limited mobility can also have negative physical consequences, such as osteoporosis, hypercalcemia, obesity, pain, and decubitus ulcers, as well as psychological consequences such as sleep disturbances and depression [12,13,14].

It is also important to consider the potential impact of blood parameters on physical mobility in HD patients. For example, high levels of inflammation, such as C-reactive protein (CRP), have been linked to decreased physical function in HD patients [15]. Similarly, low levels of hemoglobin and erythrocyte mass can also contribute to impaired physical mobility due to anemia [16].

Since only in-center HD is currently available in Slovenia, patients have to commute to dialysis facilities usually three times a week; therefore, this complex situation causes a considerable burden on the patients, patient’s relatives, and healthcare providers, especially in patients with impaired mobility. To the best of our knowledge, no study had comprehensively investigated the overall burden of limited physical mobility in prevalent HD patients. Therefore, our research aimed to fill this gap by assessing the prevalence, causes, and clinical correlations of impaired physical mobility in HD patients in Slovenia.

## 2. Materials and Methods

### 2.1. Patients and Study Design

We conducted a cross-sectional observational study of all patients from the Centre for Acute and Complicated Dialysis at the University Medical Centre of Ljubljana. We employed a consecutive enrollment approach, where all patients receiving hemodialysis at the center were enrolled. The sample size of 205 patients was deemed sufficient for our study objectives, as it allowed us to comprehensively investigate the prevalence, causes, and clinical correlates of impaired physical mobility in our specific population of hemodialysis patients in Slovenia. Data were collected through interviews based on a pre-prepared questionnaire (Appendix A), based on files filled by nephrologists and dialysis nurses, and a review of the medical history.

Included patients received hemodialysis for 4–5 h, three times per week. Dialysis procedures were performed with a standard bicarbonate-based dialysate and using a high-flux HD membrane: polyamide high-flux hemodialyzer (Polyflux 140 H, 170 H, 210 H; Gambro Dialysatoren GmbH, Hechingen, Germany) or polysulfone high-flux hemodialyzer (Fx 60, Fx 80, Fx 100; Fresenius, Bad Homburg, Germany).

Patients were classified into three mobility problem groups: (1) no or inconsiderable impairment of physical mobility, (2) minor mobility impairment, and (3) severe mobility impairment. We defined minor mobility impairment as a need for mobility aid in the form of one or two crutches or a walking frame. Severe mobility impairment was defined in three levels: being confined to bed, confined to bed but able to perform some movements, and ambulatory but dependent on the assistance of a third person for locomotion. The causes of impaired mobility were classified as neurological (ischemic brain injury, intracerebral hemorrhage, traumatic brain injury, dementia, parkinsonism…), cardiovascular (ischemic heart disease, heart failure, peripheral arterial disease…), musculoskeletal (age-related losses of muscle mass, amputation, osteoporosis with fractures, spine injury…), and others.

Data collection included demographics, dialysis vintage, leucocyte count, hemoglobin levels, thrombocyte count, serum calcium, phosphate, intact parathormone (iPTH), albumin concentrations, and CRP levels.

This study was performed in compliance with the Declaration of Helsinki (as revised in Fortaleza 2013) and was approved by the National Medical Ethics Committee (0120-280/2018); patient consent was waived due to the observational nature of this study.

### 2.2. Statistical Analysis

Statistical analysis was performed using IBM SPSS statistics program (version 28.0; Chicago, IL, USA). The normality of the data was assessed with the Kolmogorov–Smirnov test. Data are reported as a mean ± standard deviation (SD), absolute frequency, or percentage. Spearman’s correlations were used to calculate relationships between mobility problems, dialysis vintage, and blood parameters (hemoglobin, calcium, phosphate, iPTH, albumin, CRP). Additionally, correlation analysis was conducted while controlling for age. Correlation strength was interpreted as r = 0 to 0.3, or 0 to −0.3, small; 0.31 to 0.49, or −0.31 to −0.49, moderate; 0.5 to 0.69, or −0.5 to −0.69, large; 0.7 to 0.89, or −0.7 to −0.89, very large; and 0.9 to 1, or −0.9 to −1, perfect correlation [17]. Furthermore, a multivariate linear regression analysis was performed to assess the combined influence of all independent variables on the extent of mobility impairment. A *p*-value of <0.05 was considered statistically significant.

## 3. Results

### 3.1. Patients’ Characteristics

The demographic and clinical characteristics of the patients are described in Table 1.

### 3.2. Impaired Physical Mobility

Most patients showed no or inconsiderable impairment of physical mobility (122/205, 60%); details are reported in Table 2. The common causes of mobility impairment were as follows: neurological (19/40, 47.5%), cardiovascular (9/40, 22.5%), musculoskeletal (8/40, 20%), and others (4/40, 10%).

The results of Spearman’s correlations for the selected variables are presented in Table 3.

A significant, moderate positive correlation was observed between mobility problems and the age of the participants (r = 0.36, *p* < 0.001), while a significant, small positive correlation was obtained between the mobility problems and CRP (r = 0.15, *p* = 0.044). Moreover, mobility problems had a small but significant negative correlation with albumin levels (r = −0.15, *p* = 0.042). Twenty-seven percent of patients had an albumin level of 35 g/L or lower. Finally, no statistically significant correlations were found between other selected variables (dialysis vintage, hemoglobin, calcium, phosphate, and iPTH) and mobility impairments.

Furthermore, we examined the correlation between clinical parameters and mobility impairment while controlling for the age of participants (Table 4).

After controlling for the age, the analysis did not reveal any statistically significant correlations between these clinical parameters (dialysis vintage, hemoglobin, calcium, phosphate, iPTH, albumin, and CRP) and mobility impairment in the studied population.

Additionally, Table 5 presents the results of the regression analysis, highlighting the predictors of mobility impairment and their corresponding coefficients, standardized coefficients, t-values, and significance levels.

The model explains a moderate portion (13.7%) of the variance in mobility problems when controlling for multiple independent variables. The adjusted R-squared coefficient indicates that about 9.2% of this variance is explained when accounting for the number of predictors in the model. The regression model is statistically significant, as indicated by the low *p*-value (0.002). This suggests that at least one of the predictor variables significantly contributes to explaining the variance in mobility problems. Age has a significant positive effect on mobility problems, with a standardized coefficient (Beta) of 0.316. This indicates that as a patient’s age increases, mobility problems tend to increase as well. Gender also has a significant positive effect, with a Beta of 0.193. This suggests that being male is associated with higher levels of mobility problems. None of the other variables show significant effects on mobility problems as their *p*-values are greater than the significance level of 0.05.

## 4. Discussion

It is well established that impaired physical mobility is a common problem among hemodialysis (HD) patients [18], and it is associated with increased morbidity and mortality [19,20]. To our knowledge, this is the first study of the prevalence and causes of impaired physical mobility in HD patients. In our cross-sectional observational study, 60% of all patients had no or little physical impairment; however, a significant proportion (40%) showed at least some degree of mobility impairment. Nineteen percent expressed a severe mobility limitation, similar to those reported by Van Loon [21] and Shimoda [22]. Patients with severe mobility impairment are unable to live on their own since they are dependent on a third person for the majority of time. Slightly higher mobility impairment was observed in a study from 2008 [23], where 57% of older adults receiving HD had some limitations in mobility. Furthermore, we observed a significant positive correlation between CRP levels and the age of patients in relation to mobility problems, along with a negative correlation between mobility problems and albumin levels. Our findings align with a related study conducted by Hirano et al. [7], which focused on the induction phase of dialysis. They observed a decline in walking independence during this phase, with age, high Charlson comorbidity index, CRP, and emergency dialysis start to be significant predictors of decreased walking independence. While our study provides valuable insights into the prevalence and causes of impaired mobility in HD patients in Slovenia, the study by Hirano et al. emphasizes the significance of addressing mobility decline during the dialysis induction phase. Combining our findings highlights the global nature of the issue and underscores the need for comprehensive strategies to prevent and manage impaired mobility in CKD patients undergoing dialysis.

In general, high prevalence of impaired mobility in dialysis patients can be at least partially explained by illnesses with a high impact on reduced mobility: cardiovascular diseases [24,25], neurological complications [24,26], and musculoskeletal disorders [18]. In line with these findings, the most common causes of mobility impairment in our cohort were neurological (47.5%), cardiovascular (22.5%), and musculoskeletal disorders (20%). Besides chronic illnesses, impaired mobility can be a consequence of age-related losses of muscle mass or acute events [27]. As expected, the age of HD patients had a significant positive correlation, and it was as an independent predictor for mobility impairment. In our cohort, patients were relatively young (mean age 63.9 ± 15.4 years) compared to the European population, and since the median age of patients starting renal replacement therapy in Europe in 2019 was 67.9 years [28], we could expect even more patients with reduced mobility in the near future with an additional high burden on medical staff.

We performed correlation analyses between mobility impairment and some laboratory data that were likely to have an impact on mobility. We measured CRP levels, since elevated CRP is associated with poorer physical function in the elderly with various comorbidities [29]. A significant, but small positive correlation was obtained between mobility impairment and CRP levels in our patients. We also confirmed a small but significant negative correlation between mobility impairment and albumin levels. In dialysis patients, the causes of hypoalbuminemia are multifactorial—a result of imbalance between albumin loss into dialysate, catabolism, and albumin synthesis [30]. Serum albumin concentration in our cohort was 37 ± 4 g/L; however, a total of 27% of patients had serum albumin levels below 35 g/L. When controlled for the age, those two parameters were not significantly correlated. Nevertheless, other studies showed that a low level of serum albumin was an independent predictor of adverse outcomes, such as mobility impairment [31] and even mortality [32]; therefore, improving the nutritional status and albumin levels is of special importance in chronic hemodialysis patients.

Deranged calcium–phosphate metabolism is a very common finding in patients with end-stage renal disease. It has been associated with disorders of bone turnover as well as with vascular and soft tissue calcification [33]. Renal osteodystrophy may occur alongside bone pain or fractures, leading to impaired physical mobility, and vice versa, and immobility can lead to osteoporosis and hypercalcemia [34]. Vascular calcifications lead to arterial stiffening, damaged microcirculatory beds, and an increase in cardiac afterload, and contributes to the development of left ventricular hypertrophy, cardiac dysfunction, and, consequently, impaired physical mobility [35]. We found no association with parameters of CKD-mineral bone disease. That finding could be partially explained by the fact that, in our patients, calcium concentration in iPTH levels were very tightly controlled.

We expected to find an association between lower hemoglobin levels and impaired physical mobility, since common symptoms of anemia are fatigue, dyspnea, and decreased physical function. According to our results, we found no such correlation. One of the possible explanations for this unpredicted finding could be that the majority (83%) of our patients had hemoglobin levels above 100 g/L, which are associated with few specific clinical symptoms. Similarly, there was no correlation between dialysis vintage and impaired mobility, even though dialysis vintage is an independent predictor of osteoporosis [36] and is associated with somatic symptoms (e.g., fatigue) [37]. Gender also played a significant role, with male patients showing a higher degree of mobility impairment compared to their female counterparts.

Our study has several strengths: (1) Comprehensive data collection—this study collected data through interviews, medical history reviews, and questionnaire-based assessments, providing a comprehensive understanding of the patients’ mobility status and associated factors. (2) Representative sample—this study included all patients from the Centre for Acute and Complicated Dialysis at the University Medical Centre of Ljubljana, which enhances the representativeness of the findings and allows for generalization to a similar population. (3) Clear classification of mobility impairment—this study classified patients into three mobility impairment groups based on specific criteria, providing clear categories to analyze and compare the levels of impairment.

This study has five main limitations: (1) Cross-sectional design—as a cross-sectional study, the findings only provide a snapshot of the patients’ mobility and associated factors at a specific point in time. Longitudinal studies would be required to establish causal relationships and observe changes over time. (2) Limited generalizability—this study was conducted at a single medical center in Ljubljana, Slovenia, which may limit the generalizability of the findings to other populations or settings. Further research involving diverse populations would help validate the results. (3) Self-reporting bias—data collection relied on interviews and medical history reviews, which could be subject to recall bias or misreporting by patients or healthcare professionals. Objective measures or additional assessment tools could strengthen the validity of the findings. (4) Limited scope of variables—this study focused on a specific set of variables, such as demographics, dialysis vintage, blood parameters, and causes of impaired mobility. Other potential contributing factors, such psychosocial factors, were not considered, limiting the comprehensive understanding of mobility impairment in hemodialysis patients. (5) Another significant limitation of our study is the absence of a control group for comparison, particularly in terms of assessing the mobility and physical disability of HD patients versus those in the early stages of CKD. Including such a control group would have allowed for a more comprehensive evaluation of the impact of HD on physical mobility and a better understanding of how mobility changes across different stages of CKD. Future research endeavors should consider incorporating control groups to address this aspect and provide a more holistic perspective on the mobility challenges faced by CKD patients.

From our data, it is clear that the prevalence of mobility impairment is large and therefore it is important to assess and address this as a part of high-quality holistic dialysis care. The possible underlying causes of impaired mobility (neurological, cardiovascular, and musculoskeletal disorders) may be amenable to preventive interventions [38]. In addition, exercise interventions with a physical therapist or other healthcare provider should be incorporated to improve muscle strength and function—with intradialytic exercise being the first obvious opportunity, which may significantly improve physical performance (e.g., intra-dialysis exercise [39]). It is also important to address any psychological and cognitive consequences of hemodialysis treatment and intervene to limit these influences [40].

## 5. Conclusions

In conclusion, our study highlights the high prevalence of impaired mobility among chronic hemodialysis patients. Neurological, cardiovascular, and musculoskeletal diseases were identified as the most common causes of mobility limitations in this population. Only the age and male gender of the participants were found to be independent predictors of impaired mobility. Further investigations are warranted to identify additional risk factors and develop effective strategies to mitigate the burden of immobility on patients, their families, and healthcare providers. By addressing these factors, we can potentially enhance the quality of life and clinical outcomes in HD patients.

## Figures and Tables

**Table 1:** Demographic and clinical characteristics of patients. Data are presented as mean ± standard deviation (range) or percentage.

Parameter	Value
N	205
Age [years], range	63.9 ± 15.4 (24–92)
Male gender	119 (58%)
Dialysis vintage [years], range	7.3 ± 9.0 (1–44)
Comorbidities	
Diabetes mellitus	77 (38%)
Arterial hypertension	193 (95%)
Peripheral vascular disease	55 (27%)
Laboratory values	
Leucocytes (10 * 9/L)	6.4 ± 2.3
Hemoglobin (g/L)	117 ± 13
Thrombocytes (10 * 9/L)	185.4 ± 60.5
Calcium (mmol/L)	2.2 ± 0.3
Phosphate (mmol/L)	1.4 ± 0.4
iPTH (ng/L)	430.4 ± 488.1
Albumin (g/L)	37 ± 4
CRP (mg/L)	12 ± 18

Abbreviations: N, number of subjects; CRP, C-reactive protein; iPTH, intact parathormone.

**Table 2:** Prevalence of different stages of immobility (N = 205).

Physical Mobility	Value (*n* (%))
**No or inconsiderable impairment (%)**	122 (60)
**Minor mobility impairment (%)**	43 (21)
A crutch	10 (5)
Two crutches or a walking frame	9 (4)
Intermittent help of a third person	24 (12)
**Severe mobility impairment (%)**	40 (19)
Confined to bed	19 (9)
Confined to bed but able to perform some movements	19 (9)
Dependent on assistance of a third person	2 (1)

**Table 3:** Correlation coefficients between clinical and demographic parameters variables and mobility impairment.

Variable	Mobility Impairment	
	r	*p*
Age	0.36 **	<0.001
Dialysis vintage	0.01	0.922
Hemoglobin	−0.04	0.569
Calcium	0.02	0.836
Phosphate	−0.03	0.671
iPTH	−0.05	0.490
Albumin	−0.15 *	0.042
C-reactive protein	0.15 *	0.044

Note: **, significance at *p* < 0.001 level; *, significance at *p* < 0.05 level. Abbreviations: iPTH, intact parathormone.

**Table 4:** Correlation coefficients between clinical parameters variables and mobility impairment controlled for age.

Variable	Mobility Impairment	
	r	*p*
Dialysis vintage	0.065	0.375
Hemoglobin	0.093	0.207
Calcium	0.000	0.995
Phosphate	−0.039	0.593
iPTH	0.003	0.965
Albumin	−0.103	0.161
C-reactive protein	0.022	0.767

Abbreviations: iPTH, intact parathormone.

**Table 5:** Regression analysis results for mobility impairment predictors.

Independent Variable	Regression Coefficient	Standardized Regression Coefficient Beta	t	*p*	95% CI	Coefficient of Determination R^2^	F	*p*
Age	0.013	0.316	4.077	<0.001	0.007–0.019	0.137	3.018	0.002
Gender	0.238	0.193	2.601	0.010	0.057–0.418			
Dialysis vintage	0.007	0.099	1.338	0.183	−0.003–0.017			
Hemoglobin	0.003	0.075	1.017	0.311	−0.003–0.009			
Calcium	0.091	0.032	0.416	0.678	−0.340–0.522			
Phosphate	0.000	0.000	0.005	0.996	−0.176–0.177			
iPTH	−1.423 × 10^−5^	−0.010	−0.136	0.892	0.000–0.000			
Albumin	−0.024	−0.147	−1.688	0.093	−0.051–0.004			
C-reactive protein	−0.002	−0.047	−0.557	0.579	−0.007–0.004			

Abbreviations: iPTH, intact parathormone; CI, confidence interval.

## Data Availability

The datasets generated and/or analyzed during this study are available from the corresponding author upon reasonable request.
